# Hybrid Capsule Network for precise and interpretable detection of malaria parasites in blood smear images

**DOI:** 10.3389/fcimb.2025.1615993

**Published:** 2025-08-08

**Authors:** Bader Alawfi

**Affiliations:** Department of Clinical Laboratory Sciences, College of Applied Medical Sciences, Taibah University, Madinah, Saudi Arabia

**Keywords:** malaria detection, Capsule Network, Hybrid CapNet, parasite classification, life cycle stage recognition, blood smear microscopy

## Abstract

**Introduction:**

Rapid and precise malaria diagnosis is critical in resource-constrained settings to enable timely treatment and reduce mortality. Existing convolutional neural network (CNN) and capsule network hybrids, although effective, often suffer from high computational demands and limited generalizability across datasets.

**Methods:**

We propose Hybrid Capsule Network (Hybrid CapNet), a lightweight architecture combining CNN-based feature extraction with dynamic capsule routing for accurate parasite identification and life-cycle stage classification. A novel composite loss function—integrating margin, focal, reconstruction, and regression losses—was employed to enhance classification accuracy, spatial localization, and robustness to class imbalance and annotation noise. The model was evaluated on four benchmark malaria datasets (MP-IDB, MP-IDB2, IML-Malaria, MD-2019) and assessed for both intra- and cross-dataset performance.

**Results:**

Hybrid CapNet achieves superior accuracy with significantly reduced computational cost (1.35M parameters, 0.26 GFLOPs), rendering it suitable for mobile diagnostic applications. Experimental results demonstrate up to 100% accuracy in multiclass classification and consistent improvements over baseline CNN architectures in cross-dataset evaluations. Grad-CAM visualizations confirm that the model focuses on biologically relevant parasite regions, validating interpretability.

**Discussion:**

The proposed framework delivers a pragmatic and interpretable solution for malaria diagnosis, balancing high accuracy with minimal computational requirements, and demonstrates strong potential for deployment in real-world, resource-limited clinical environments.

## 1 Introduction

Malaria is considered one of the most ancient and severe infectious diseases in human history Paica et al. (2023). It is transmitted to humans through the bite of a female Anopheles mosquito and is caused by one of five Plasmodium species—Knowlesi, Ovale, Malariae, Vivax, or Falciparum Prabhu et al. (2021). In 2022, the WHO anticipated 249 million malaria cases in 85 countries and 608,000 fatalities. Interestingly, Africa, where these diseases originally appeared and account for 94% of global malaria incidence and deaths, bears a significant burden. Falciparum, the deadliest plasmodium species, is more common in Africa, although Vivax is found elsewhere Organization, W. H (2023). Symptoms such as headache, fever, and fatigue typically emerge 10–15 days after being bitten by a mosquito carrying the Plasmodium parasite. If left untreated, it can progress to acute respiratory distress, coma, seizures, organ failure, and ultimately death.

A carrier mosquito introduces the malaria parasite, which uses red blood cells to live and reproduce. There are four species of malaria parasites—P. malariae, P. ovale, P. vivax, and P. falciparum—with the latter two being the most commonly encountered Price et al. (2020). Malaria infects aged RBCs and young RBCs of the vivax type Neveu and Lavazec (2021). Different species exhibit distinct lifespans and maturation periods; some can remain dormant for weeks or even cause a relapse after the initial infection Bousema and Drakeley (2011). Thus, infected cells must be diagnosed for the type of infection and the specific parasite involved. Each malaria parasite progresses through four stages: gametocyte, ring, trophozoite, and schizont Adegoke et al. (2022); Nasir et al. (2024c). Identifying the particular stage of the parasite is essential for effective treatment, as different phases demand timely and targeted intervention. The trophozoite and ring stages of the parasite are most visible in people; therefore, detecting the disease early improves patient survival and organ safety Kochan et al. (2021). In addition, malaria parasite identification and detection are less studied than life cycle stage classification.

Malaria’s early symptoms and signs are like those of typhoid. Thus, laboratory identification is needed to treat and prevent it Cerilo-Filho et al. (2024). Thin and thick blood smear microscopy remains the most reliable and widely used method for diagnosing malaria. To detect and identify malaria parasites, a patient’s blood is spread on a glass slide, stained to highlight the parasites by color, and then examined under a microscope by a skilled microscopist to determine both their presence and species within red blood cells. This method is labor-intensive, time-consuming, and subjective, with its accuracy heavily dependent on the microscopist’s expertise—an expertise often lacking in malaria-endemic regions Mbanefo and Kumar (2020); Tehsin et al. (2024b).

Current malaria diagnosis involves mechanically detecting parasites in red blood cells in blood slides. If parasites are detected, the type, life cycle stage, and number of infected RBCs are studied White (2022). The infrastructure and pathologists’ skills determine accuracy. High-volume samples might be evaluated hundreds of times without a qualified pathologist, leading to misdiagnosis, especially in overcrowded medical institutions in flood-stricken areas of Pakistan Mohammed and Abdelrahman (2017); Tehsin et al. (2024c). The test procedure takes time and costs people and money. If they don’t increase errors, speed up findings, and cost less, computer-aided diagnostic (CAD) systems can reduce this strain. Traditional image processing, based on cell image intensity values and increased morphological features, has been used to detect malaria parasites and their types earlier Punitha et al. (2017). More advanced machine learning and deep learning architectures, such as convolutional neural networks (CNNs), are favored due to their ability to deliver superior diagnostic performance Razzak (2015); Yousafzai et al. (2024). Like any deep learning architecture, these require strong hardware and network coverage. CAD systems are unviable in nations with significant economic disparities between rural and remote areas, as well as inadequate computer hardware and internet connectivity. We require a deep learning method that is computationally efficient, portable on mobile devices, and does not rely on the internet or other digital tools.

Various CAD systems utilizing conventional image processing or machine learning techniques have been proposed for malaria detection. These approaches, while beneficial, often rely heavily on manually built features, color thresholding, or morphological indicators, which restrict their applicability across datasets with varying staining protocols or lighting conditions. With the emergence of deep learning, CNNs have gained prominence in malaria classification due to their exceptional capabilities in representation learning. Nonetheless, CNNs are intrinsically constrained in their ability to capture hierarchical pose relationships due to max-pooling layers, which frequently leads to a loss of spatial context—essential in blood smear research, where parasite morphology exhibits subtle variations. Capsule Networks (CapsNets), designed to maintain spatial hierarchies, have demonstrated potential in medical imaging applications. Nonetheless, independent CapsNet models often demonstrate inadequate scalability and require meticulous routing strategies that can be computationally demanding. Recent hybrid models that integrate CNNs with Capsule layers largely mitigate these challenges but continue to have optimization instability and constrained interpretability. Moreover, the majority of these studies focus on binary categorization (infected versus uninfected) and overlook the classification of life cycle stages, which is crucial for effective treatment planning and management. The identified limitations drive the creation of Hybrid CapNet, a lightweight and interpretable architecture featuring an innovative composite loss function designed to ensure precise classification, localization, and reconstruction while maintaining computational efficiency and robustness across various datasets.

The research presents Hybrid CapNet, an innovative architecture that accomplishes precise malaria parasite identification and various dataset classification tasks. The model performs dual diagnostic duties by identifying parasite types and life cycle stages, enabling users to obtain enhanced diagnostic information beyond binary classification. The architecture integrates convolutional layers for feature extraction with capsule layers that preserve spatial hierarchies, enhancing resilience to morphological and orientation fluctuations in microscopic smear images. The innovative loss function integrates margin loss with reconstruction loss, focused classification loss, and offset regression loss to facilitate concurrent learning of diagnostic precision, spatial accuracy, and enhanced noise immunity. The model demonstrates its effectiveness across four benchmark datasets—Malaria-Detection-2019, IML-Malaria, MP-IDB2, and MP-IDB—through both in-dataset and cross-dataset evaluations, confirming its superiority over existing CNN-based models in terms of accuracy and generalization performance. This architecture employs a lightweight design featuring 1.35 million parameters and 0.26 GFLOPs of operations, which facilitates deployment on mobile diagnostic devices in resource-constrained environments. Interpretability is ensured by Grad-CAM visuals that illustrate the model’s focus on clinically significant regions within the stained blood smear images. The collaborative research yields a reliable AI-driven diagnostic tool for field malaria assessment, offering both efficacy and clarity.

This work begins by presenting the shortcomings in malaria diagnosis, followed by an examination of deep learning methodologies and their existing constraints. The methodology outlines the proposed structure of the Hybrid CapNet model, the datasets used, the preprocessing techniques employed, the training methodology, and the implementation of loss functions. The experimental findings encompass performance assessments of four datasets, cross-dataset validations, and Grad-CAM interpretation methodologies. The research article concludes with a summary of the obtained data and an analysis of potential future endeavors.

## 2 Related literature

Numerous computer-aided diagnostic (CAD) systems have been developed to classify malaria parasites from blood smear images Fatima and Farid (2020). The binary classification of malaria Rahman et al. (2019) uses patient blood slide images to categorize blood cells as infected or not. Most available malaria datasets include only healthy and infected labels, as well as blood slide images Rajaraman et al. (2018a). Conventional malaria classification is based on morphological properties of infected blood cells Savkare and Narote (2015), image capture process improvement Fn et al. (2016), and cell size and image intensity information Kareem et al. (2012). Some studies have evaluated the quantity of healthy red blood cells and malaria-infected cells using whole-image color spaces to determine whether they are healthy or infected May et al. (2013).

In contrast, pixel discrimination was used to distinguish malarial cells Roy et al. (2018).In Arco et al. (2015), histogram equalization and connected component analysis are used to estimate malaria parasite density. However, traditional image processing techniques tend to be slow and rely heavily on dataset specific parameters, such as image intensity and intense color contrasts typically introduced through staining. We employed both machine learning and deep learning architectures to enhance image classification accuracy and accelerate the diagnostic process. Stacking CNNs to perform binary classification of malaria Umer et al. (2020); Yousafzai et al. (2025b) or more complex deep learning architectures with greater accuracy Gautam et al. (2020) is a common approach in machine learning. Neural networks have also been pre-trained to improve binary malaria classification and automatic malaria patient identification in blood slide images Imran et al. (2022).

Several datasets with multiclass labels for malaria parasites are available Loddo et al. (2019); however, the subject is not well-explored. Kassim et al. (2021) classified thick smear images of P. vivax and P. falciparum. Recently published datasets included malaria parasite type with blood slides and multiclass labels for life cycle stage Arshad et al. (2022). They propose using pre-trained neural network architectures based on deep learning to classify and segment the phases of the malaria life cycle. Loddo et al. (2022); Yousafzai et al. (2025a) used P. Falciparum malaria type blood slide images for multiclass life cycle stage classification. Another malaria life cycle classifier identifies the parasite in the trophozoite, schizont, and ring stages Abbas and Dijkstra (2020). Recent research on the classification of the Plasmodium parasite life cycle, employing data from Hospital Universiti Sains Malaysia, has utilized AlexNet and GoogleNet. GoogleNet and AlexNet achieved test accuracies of 89.1% and 91.1%, respectively Azhar et al. (2023). In a separate study, Deep Neural Networks classified Plasmodium into four life cycle stages—gametocyte, trophozoite, schizont, and ring—achieving an accuracy of 87.95% with EfficientNet-B7, outperforming other models Araujo et al. (2023); Tehsin et al. (2025).

To minimize image inversion blur, the proposed Wiener filter reduces additive noise, and the Median filter is added for impulsive noise, similar to MOHD AZIZ (2013). Arco et al. (2015) successfully removed Gaussian noise from images using a Gaussian filter. Das et al. (2015) developed the Geometric Mean Filter to preserve the edges of microscopic images while effectively reducing Gaussian noise. Savkare and Narote (2015) developed a Laplacian filter for enhancing edges and smoothing images, while Soni et al. (2011) employed the SUSAN filter to preserve image quality and structural details. Díaz et al. (2009) applied a low-pass filter that averages image pixel intensities to eliminate high-frequency components. In contrast, Suradkar (2013) introduced adaptive local histogram normalization to preserve contrast in low-resolution images. Abbas and Mohamad (2013) recommended histogram matching to equalize pixel intensities, while various contrast enhancement techniques have been consistently used to improve image clarity. Lighting correction is often done using the Grey World assumption Liu et al. (2020).

In experiments using the standard NIH dataset, VGG16 identified malaria 95.96% accurately Huq and Pervin (2020); Nasir et al. (2024b). The original set of 27,556 images was resized to 224 × 224 pixels. A customized sequential CNN achieved an F1 score of 95.90%, sensitivity of 94.70%, and accuracy of 92.70% Rajaraman et al. (2018b). On the same malaria dataset, ResNet50 attained 95.40% accuracy Reddy and Juliet (2019). A bespoke CNN model with five convolutional and pooling layers reached 96.33% accuracy and an F1 score of 96.82% Maqsood et al. (2021). Additionally, we developed a hybrid platform designed to reduce both structural and empirical risks, achieving 93.44% sensitivity and 93.13% accuracy Var and Tek (2018). Another Capsule Network (CapsNet) hybrid screening method can identify and pixel label (segmenting) malaria parasite-infected RBCs with 98.70% accuracy Maity et al. (2020). Modified YOLOv4 and YOLOv3 models achieved accuracies of 96.14% and 95.46%, respectively, on a public malaria dataset Abdurahman et al. (2021). More recently, YOLOv5 and Darknet-53 were used to detect Plasmodium falciparum life stages, attaining accuracies of 96.02% and 95.20%, respectively Zedda et al. (2022). However, earlier work utilizes CNN models to detect parasite images in natural samples. In contrast, medical images show parasite malaria infection patterns and textures that limit model performance.

Recent advancements in deep learning-based malaria diagnostics have yielded numerous high performing architectures, especially post-2022. Kumar et al. (2024); Malik et al. (2024) introduced an Inception-based Capsule Network that attained over 95% accuracy in the binary categorization of parasitized and uninfected red blood cells. Their model, albeit useful, was restricted to coarse-level judgments and failed to consider parasite stage or localization. Mujahid et al. (2024) developed an EfficientNet-based CNN classifier, employing five-fold cross-validation, which achieved an accuracy of 97.57% on red blood cell pictures. This model mainly concentrated on cell-wise classification and did not investigate cross-dataset generalization or spatial context. Nettur et al. (2025) introduced UltraLightSqueezeNet, a parameter-efficient network that attained accuracy rates of 96.6% to 97.1% while utilizing considerably fewer parameters than conventional models, rendering it appropriate for embedded platforms, albeit deficient in explainability and stage differentiation.

Additionally, Ali et al. (2024); Tehsin et al. (2024a) presented M2ANET, a mobile-optimized deep learning architecture that integrates MobileNet inverted residual blocks with a mobile self attention mechanism. Their architecture exhibited competitive accuracy while being optimized for real-time field diagnostics deployment. Furthermore, Abbas and Dijkstra (2020) utilized random forest classifiers to identify Plasmodium falciparum in Giemsa-stained thin smear images, presenting stage classification outcomes with commendable efficacy. However, the traditional nature of their methodology limited its scalability and profound representational capacity. Chaudhry et al. (2024) introduced a CNN-based approach for classifying malaria life cycle stages from annotated blood smear pictures, attaining an accuracy of around 80%. Nevertheless, the system was trained exclusively on a singular dataset and failed to integrate localization cues or account for model uncertainty. Madhu et al. (2023) developed an Inception-V3-based capsule network for binary classification of malaria-infected cells, achieving 99.35% accuracy and 99.73% AUC on the NIH dataset. Their model combined multi-scale feature extraction with capsule routing to preserve spatial hierarchies. Though effective, it focused solely on binary tasks without addressing stage classification, interpretability, or cross-dataset validation.

Notwithstanding these contributions, numerous issues endure. The majority of the previously stated models focus exclusively on binary detection tasks and are generally validated on a single dataset, such as the NIH Malaria Cell Image dataset. They frequently exhibit inadequate mechanisms for spatial localization, neglect multiclass life cycle stage classification, and are deficient in aspects of interpretability essential for clinical integration. Moreover, although several models reduce the parameter count or inference duration, they seldom provide a comprehensive solution that concurrently addresses classification, localization, robustness to class imbalance, and explainability. Conversely, our proposed Hybrid CapNet enhances the current state of the art by integrating an innovative composite loss function comprising margin, focal, reconstruction, and regression losses to augment diagnostic accuracy, spatial localization, and model resilience in the presence of noise and imbalance. Our model, with merely 1.35 million parameters and 0.26 GFLOPs, achieves state-of-the-art accuracy while facilitating real-time inference on low-resource devices. In contrast to previous techniques, Hybrid CapNet is comprehensively verified on four public datasets, exhibiting consistent generalization across various imaging settings and annotation methodologies. Its interpretability is further augmented by Grad-CAM-based visuals, which emphasize contaminated areas and bolster physician confidence. Hybrid CapNet is established as a robust, scalable, and transparent approach for classifying malaria parasites and their stages across various clinical contexts.

## 3 Materials and methods

The comprehensive description of the proposed methodology is located in Section 3. The study employs four distinct datasets: MP-IDB, MP-IDB2, IML-Malaria, and Malaria-Detection-2019, detailing their staining techniques, image resolutions, and annotation criteria in Section 3.1. The model training procedure involves normalization and subsequent image augmentation, as detailed in Section 3.2. Section 3.3 illustrates how Hybrid CapNet. derived from original Capsule Network Sabour et al. (2017), integrates diverse components, routing mechanisms, and tailored loss functions to formulate its architecture and attain classification, localization, and reconstruction capabilities. The training algorithm outlined in Section 3.4 is determined by hyperparameter configurations, routing iterations, and the optimization technique. Each subsection offers fundamental principles for constructing an accurate, interpretable, and efficient framework for analyzing blood smear images.

### 3.1 Datasets

The MP-IDB dataset Loddo et al. (2019); Nasir et al. (2024a), available at the Centre Hospitalier Universitaire Vaudois (CHUV), offers free access to images of mosquito parasites captured using an optical laboratory microscope equipped with an integrated camera setup. In total, there are 229 full-slide blood images representing four malaria parasite types: P. ovale, P. malariae, P. vivax, and P. falciparum. These complete-slide PNG images possess a 24-bit color depth and a resolution of 2592 × 1944 pixels. The parasites are categorized into four distinct stages, which include schizont development, ring phases, trophozoite growth, and gametocyte growth. MP-IDB2 provides PNG files containing images of cellular parasites of varying resolutions. The illumination parameters and background stability exhibit inconsistent levels, as well as varying border dimensions, despite the fact that these images were acquired using a single microscope.

Standard components for the classification of malaria life cycles in thin blood smear images generated from Giesema-stained whole blood slides are included in the IML-Malaria Arshad et al. (2022). The public was introduced to a dataset in 2021 that provides 345 microscopic images. Each image in the dataset is in JPG format and has a resolution of 1280 × 960 pixels. JSON array data is present in each image of the dataset, and a distinct annotation file is also included. “image name” and “objects” are the two primary elements of the JSON objects. The “type” values in the objects array correspond to the cell categories in each image, as indicated by the bounding box outline. The publicly available dataset includes annotations for various blood cell types, such as “difficult,” “gametocyte,” “trophozoite,” “schizont,” “ring,” and “red blood cell,” among others.

**Figure 1:**
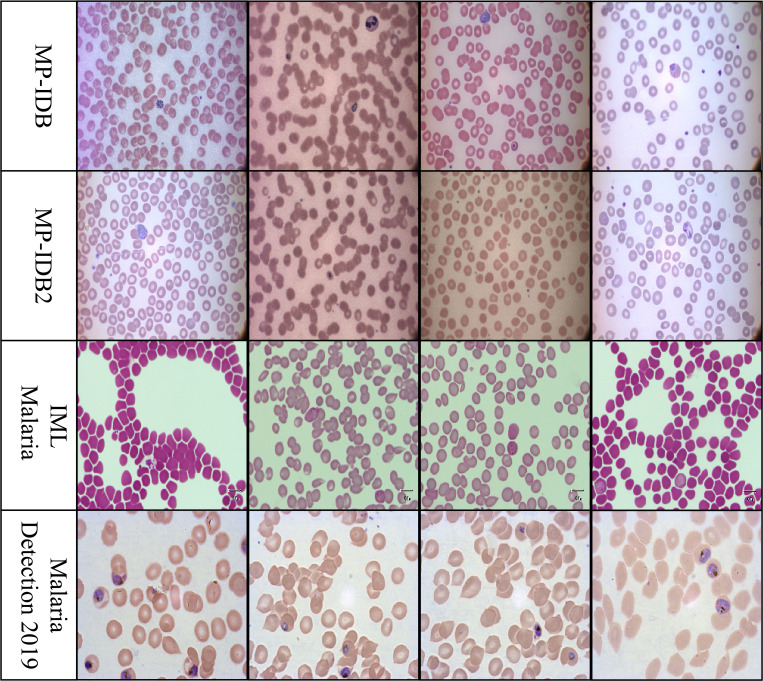
Representative cell images from each dataset used in this study: MP-IDB, MP-IDB2, IMLMalaria, and MP-IDB2, illustrating differences in staining, resolution, and parasite appearance across data sources.

The Malaria-Detection-2019 dataset is promptly accessible via the internet Abbas and Dijkstra (2020); PP and Tehsin (2025). Triple comprises 883 full-body slide images of malarial parasites that have been stained with Giesema. The PNG-type images in this collection have a resolution of 1382–1030 pixels. The life cycle stage of the specimens is determined by the inclusion of supplementary labels in the dataset. The dataset includes eight possible labels: garbage, white blood cells (WBC), mid trophozoite (MT), early trophozoite (LR-ET), segmenter (Seg), ring (R), late schizont (Lsch), and early schizont (Esch). The authors encountered significant challenges in distinguishing between all eight periods due to the early and late labels. To evaluate rings, trophozoites, and schizonts, the research group simplified three biological assessment stages from the original eight phases. We will adhere to the same evaluation system throughout our investigation. Sample images from each dataset are shown in **Figure 1**.

### 3.2 Preprocessing

The MP-IDB dataset was partitioned into three subsets for training, validation, and testing, with parasite classes allocated as 66% for training and 17% each for validation and testing. The IML subsets were constructed by the researchers using division methods that adhered to the guidelines established by the authors Arshad et al. (2022). The proposed tasks obtain consistent results by utilizing mean and standard deviation normalization on all images. We employed full-slide blood smear images to classify various malaria parasite types, as illustrated in the image examples from the previous section. Infected labeled cells were generated by extracting cell crop segmentations from the annotation files of the Malaria-Detection2019 and IML-Malaria datasets. The life cycle stage classification information from the MPIDB2 dataset was pre-provided by cropping the infected labeled cells. All images used for classifying malaria life cycle stages in MP-IDB2, Malaria-Detection-2019, and IML-Malaria exhibit symmetrical properties.

Because these classes were disproportionately represented in the training datasets, the networks were at a high risk of conforming better to them. To resolve this issue, various augmentation techniques were implemented. In our study, we applied horizontal and vertical flip augmentations, along with the random pad and random crop methods. These modifications were individually incorporated into each batch sequence, with a 50% likelihood of being implemented. After undergoing augmentations, the input images must be resized to 224/299 pixels for all other architectures. Prior to processing, the input framework necessitates that the images be transformed into tensors. **Table 1** presents statistical information on the full-slide images, cropped images, and augmented images across all datasets utilized in this study.

**Table 1:** The data utilization statistics for malaria parasite-type and life cycle stage classification processes.

Dataset	Full	Crops	Augmented	Classes	Resolution
MP-IDB	210	–	105	4	224
MP-IDB2	–	1361	680	4	224
IML_Malaria	–	427	213	4	224
md-2019	–	1361	680	3	224

To alleviate the effects of class imbalance—especially for infrequent parasite kinds and less commonly observed life stages—we implemented focused data augmentation techniques. These encompassed horizontal and vertical flips, random padding, and cropping, with an increased likelihood of implementation for underrepresented classes. The synthetic augmentation of the minority classes facilitated the equalization of their contribution during training. Additionally, during batch construction, we implemented balanced sampling to guarantee that each mini-batch included representative samples from all classes, thereby mitigating the model’s propensity to overfit to predominant classes.

### 3.3 Hybrid CapsuleNet

**Figure 2:**
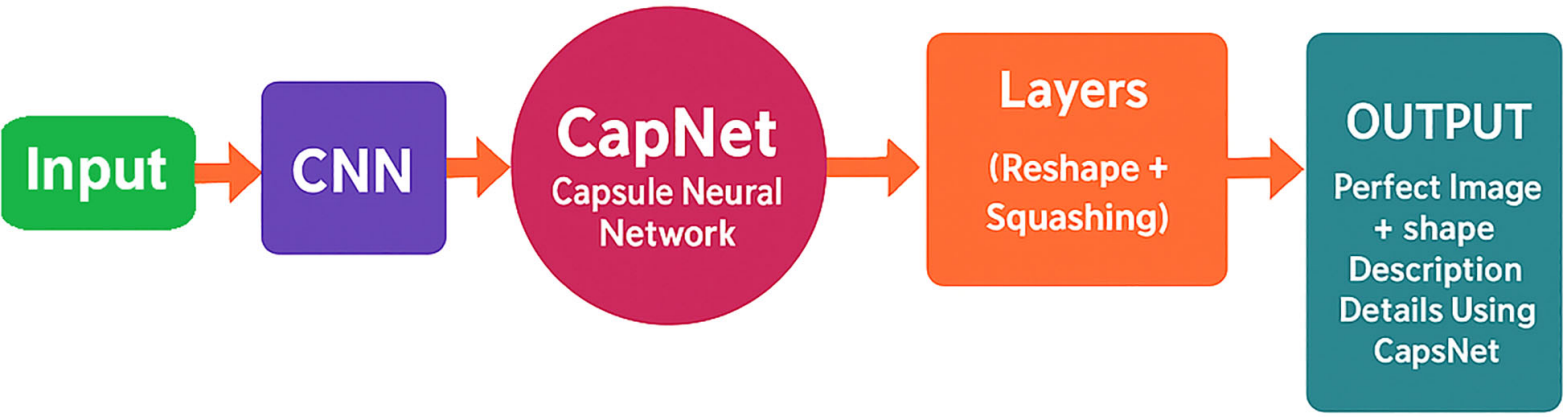
Hybrid CapNet architecture for the detection of malaria parasites. The model processes a preprocessed input image (224×224 RGB) and then performs convolutional feature extraction, integrating capsule layers with dynamic routing, and utilizes multi-loss optimization for classification, localization, and reconstruction.

The hybrid CapNet model exhibited outstanding performance when applied to the analysis of microscopic blood smear images for malaria diagnosis. Several critical procedures must be implemented prior to the commencement of model training to optimize its functionality. Image enhancement is considered one of the most sophisticated techniques by experts due to its ability to incorporate new training data by modifying the original images. This technique enhances the operational efficacy of the model when it is presented with a variety of data types. Rotational and flipping procedures, scale methods, cropping, and noise addition to images are all included in the collection of image enhancement techniques Sabour et al. (2017). Setting all pixel values in each image to adhere to a standard measurement ratio is the process of standardizing images. The impact of variations in illumination, contrast, and color on model performance is mitigated by standardization techniques. Normalization necessitates the integration of contrast expansion applications and normalization procedures combined with averaging subtraction. The distribution of the analysis data for malaria diagnosis is unbalanced, as images depicting ailments are less frequently represented than those without disease Alqudah (2020). The combination of oversampling and undersampling techniques facilitates class allocation for this issue. Image normalization encompasses a variety of tasks, including the correct resizing of images, the conversion of images to grayscale, and the enhancement of contrast to improve their quality. The methods are effective in reducing visual disturbance and irrelevant information in images, thereby rendering them suitable for model training. Image augmentation, normalization, and class evaluation must be implemented as part of an integrated solution to optimize hybrid CapNet operation for malaria diagnosis. The strategies improve the quality and diversity of the data used in the working out process while simultaneously reducing the reliance on changes in the prediction model’s input theory. The experimental method is illustrated in **Figure 2**, where as all definations of symbols and variables are presented in **Table A1** in **Appendix A**.

The actual preprocessing steps used in this study include RGB normalization, resizing all images to 224 × 224, contrast-limited adaptive histogram equalization (CLAHE) for local contrast improvement, and targeted augmentation (flipping, cropping) to address class imbalance. These are integrated directly into our training pipeline. When feasible, the data normalization process must be executed in conjunction with any necessary data supplements. The ConvNet-based feature extraction approach replaces traditional fully connected layers with convolutional operations to preserve spatial hierarchies and reduce parameter complexity. While sustaining the final classification stages, CNN maintains its capacity to detect significant spatial information through this technique. The Capsule Layer Integration approach substitutes one or more capsule layers for all connected layers within the CNN. Each capsule employs vector representation in this layer to communicate the instantiation parameters of the corresponding entity, including its shape and size. The implementation of dynamic transit between capsule layers facilitates the development of strong relationships between entities. Dynamic routing enables capsules in one layer to transmit information that impacts capsules in subsequent layers. CapNets employ this capability to enhance the generation of their spatial hierarchy. To enhance readability, we simplified and standardized subscripts across all equations. For instance, *c_ij_* now consistently refers to the coupling coefficient between capsule *i* and capsule *j*, and *u_ij_* denotes the predicted output from capsule *i* to capsule *j*. The convolutional output is flattened into a feature vector of dimension *V_f_*, which serves as input to the primary capsule layer. Here, *V_f_* denotes the total number of flattened features after convolution and pooling operations, calculated in Equation 1 as:


(1) $$V_{f}=\frac{\left(W_{i}-K_{s}+2P_{s}\right)}{S_{s}}+1$$

The vector size *V_f_* is calculated using the usual convolutional output formula, with kernel size, stride, and padding chosen to preserve adequate spatial resolution. The convolutional depth and filter dimensions were empirically refined to achieve compactness and precision, employing 3×3 kernels with progressively increasing channels across layers for hierarchical representation learning. The computation entails the padding value *P_s_* with a stride of *S_s_*, the convolution kernel size represented by *K_s_*, and the image width denoted by *W_i_*. While maintaining the vector direction, the squashing function *v_k_* non-linearly transforms capsule outputs into unit vectors is calculated in Equation 2 as:


(2) $$v_{k}=\frac{∥z_{k}{∥}^{2}}{1+∥z_{k}{∥}^{2}}\cdot \frac{z_{k}}{\left|\left|z_{k}\right|\right|}$$

Where, *z_k_* is input to capsule *k* and *v_k_* is output of capsule *k*. To encourage capsules to encode spatial and contextual information, a reconstruction loss *L_r_* is introduced, typically based on pixel-wise reconstruction using cross-entropy is calculated in Equation 3 as:


(3) $${ℒ}_{\text{recon}}=-\frac{1}{N}\sum_{n=1}^{N}\left[y_{n}\text{log }({\hat{y}}_{n})+\left(1-y_{n}\right)\text{log }(1-{\hat{y}}_{n})\right]$$

Here, $y\in {\mathbb{R}}^{C}$ denotes the one-hot ground truth label vector, $\hat{y}\in {\mathbb{R}}^{C}$ is the predicted class probability distribution and index *N* refers to the number of capsules in the lower layer. CapNet dynamically computes relationships between capsule pairs using transformations. For any capsule *j*, the transformation log relationship *R_ij_* between it and capsule *i* is calculated in Equation 4 as:


(4) $$R_{ij}=\text{log }(T_{ij})=v_{j}\cdot v_{i}+b_{j}+b_{i}$$

here, *T_ij_* is the transformation matrix between capsule *i* and *j* and *b_j_* and *b_i_* are bias terms for respective capsules, $v_{j}\in {\mathbb{R}}^{d}$ is the output vector of capsule *j* after applying the squash function, capturing both the probability and instantiation parameters of the detected entity. The Capsule Potential function *ϕ* aggregates transformed outputs and evaluates their alignment is calculated in Equation 5 as:


(5) $$\text{Φ}=\sum_{j=1}^{u}\sum_{i=1}^{u}e^{T_{ij}}{\left(v_{j}^{k}\cdot v_{i}+b_{j}+b_{i}-\text{log }(T_{ij})\right)}^{2}$$

*U* denotes the total number of capsules. The routing mechanism is governed by a margin loss function *L_m_*, ensuring strong agreement among capsules is calculated in Equation 6 as:


(6) $$L_{m}=\left(1-t_{k}\right)\cdot \text{max }{\left(0,∥v_{k}∥-m\right)}^{2}+t_{k}\cdot \text{max }{\left(0,m-∥v_{k}∥\right)}^{2}$$

here, *t_k_* is a indicator variable (1 if class *k* is present, 0 otherwise) and *m* is the margin threshold. The summation is taken over all lower-level capsules *i* = 1 to *N*, where *N* is the total number of capsules in the preceding layer. The SoftMax probability *P_ij_* for capsule *j* given input from capsule i is calculated in Equation 7 as:


(7) $$P_{ij}=\{\begin{array}{ll} \frac{e^{l_{ij}}}{\sum_{j}e^{l_{ij}}}, & \text{if}\;l_{ij}<l_{\text{max}}\\ 1, & \text{otherwise} \end{array}$$

here, *l_ij_* is the logit score and *l_max_* is the threshold. Pooling operations split features into smaller regions. Routing logits are normalized using a capped softmax to limit unstable coupling and ensure balanced agreement across capsules. Pooling size and stride were tuned to retain spatial integrity while reducing dimensionality. Alternative normalization schemes could enhance stability but were avoided here to preserve the model’s lightweight design. The number of pooled feature maps *Q* is determined in Equation 8 as:


(8) $$Q=\frac{q-K_{f}+2R_{s}}{S_{p}}+1$$

here, *q* is feature map dimension, *K_f_* is pooling kernel size, *R_s_* is resize padding and *S_p_* is pooling stride. The internal dynamics of CapNet also follow gated memory mechanisms similar to LSTM, which includes cell update $C_{t}=g\left(a_{t},C_{t-1}\right)$ , input gate $I_{t}=\sigma \left(W_{a}a_{t}+W_{h}h_{t-1}+W_{m}m_{t-1}+b_{i}\right)$ , forget gate $F_{t}=\sigma \left(W_{f}a_{t}+W_{hf}h_{t-1}+W_{mf}m_{t-1}+b_{f}\right)$ , memory gate $M_{t}=F_{t}☉m_{t-1}+I_{t}☉\text{tanh }(W_{m}a_{t}+W_{hc}h_{t-1}+b_{m})$ , output gate $O_{t}=\sigma \left(W_{o}a_{t}+W_{ho}h_{t-1}+W_{mo}m_{t-1}+b_{o}\right)$ and image activation $I_{m}=O_{t}☉\text{tanh }(m_{t})$ . Max pooling was utilized just in the initial layers to diminish feature map dimensions while preserving prominent local features. The ReLU activation function introduced non-linearity while maintaining spatial consistency. Capsule layers subsequently functioned on these processed features to capture pose and part-whole relationships, preserving spatial hierarchy.

To facilitate deeper routing, the network applies transformation and rotation steps across routing layers as calculated in Equation 9:


(9) $$\begin{array}{l} v_{z+1}=W_{q}\left(H_{t}+u_{z}\right)\\ H_{t}=\sigma \left(W_{q}u_{z}\right) \end{array}$$

The final CapNet activation function with sigmoid classification loss is defined as in Equation 10:


(10) $$L_{a}=-\frac{1}{n}\left(\sum_{i}\text{log }(H_{i})+\sum_{i}\text{log }(1-H_{i})\right)$$

Weight initialization, regularization, and routing update equations are as follows in Equation 11:


(11) $$\begin{array}{l} \text{Initialize weights: }W_{ij}=0\;\forall i,j\\ \text{Softmax coupling coefficient: }C_{i}=\mathrm{Soft}\max(W_{i})\\ \text{Weighted output vector: }{\text{S}}_{j\;}=\sum_{i}C_{ij}\;\cdot \;{\hat{u}}_{j∥i}\\ \text{Squashing output vertor:}{\text{ v}}_{j}=\frac{∥S_{j}{∥}^{2}}{1+∥S_{j}{∥}^{2}}\;\cdot \;\frac{S_{j}}{∥S_{j}∥}\\ \mathrm{L2 regularization:}L_{2}=\sum_{j=1}^{T}W_{j}^{2}\\ \text{Routing update:}\;W_{ij}={\hat{u}}_{j|i}\;\cdot \;{\text{v}}_{j} \end{array}$$

Each of these operations is instrumental in constructing an interpretable, high-accuracy model for malaria classification. Through the integration of dynamic routing, reconstruction loss, and squashing, as well as margin-based classification, the hybrid CapNet architecture achieves dependable learning from the complexity of medical images. The model’s classification accuracy is enhanced by the implementation of numerous parameter refining processes and routing iteration adjustments.

To improve both classification reliability and localization precision in malaria parasite detection using the Hybrid CapNet, we extend the standard capsule loss framework by incorporating advanced loss design principles, which ensures that the model is optimized not only for classifying red blood cells as infected or healthy but also for accurately detecting and localizing the infected regions within a smear image. In the Hybrid CapNet, part of the capsule output is responsible for capturing instantiation parameters that represent spatial transformations — such as the position, scale, and orientation of the parasite within the red blood cell. To evaluate the accuracy of these parameters, we define a regression loss, denoted as ${ℒ}_{\text{offset}}$ using the Smooth L1 loss function. Let $\delta_{i,\left(x,y\right)}^{\text{true}}$ be the actual transformation offset for capsule i at spatial location (x,y) and $\delta_{i,\left(x,y\right)}^{\text{pred}}$ be the predicted offset from the capsule’s output. Each offset vector comprises four elements: horizontal and vertical position deltas and scaling factors for width and height. The Smooth L1 loss for each component is defined in Equation 12 as:


(12) $${ℒ}_{\text{offset}}=\sum_{i}\sum_{d\in \left\{x,y,w,h\right\}}ℋ\left(\delta_{i,\left(x,y\right)}^{\text{true}}-\delta_{i,\left(x,y\right)}^{\text{pred}}\right)$$

where ℋ(z) is in Equation 13 as:


(13) $$ℋ\left(z\right)=\{\begin{array}{ll} 0.5z^{2}, & \text{if}\;\left|z\right|<1\\ \left|z\right|-0.5, & \text{otherwise} \end{array}$$

This loss ensures that the capsule network learns to regress to accurate instantiation parameters for parasite localization while remaining robust to annotation noise and outlier errors commonly seen in medical image datasets. Malaria diagnosis from blood smear images presents an inherent class imbalance problem, where the number of healthy cells typically far exceeds the number of infected cells. To counteract this, the CapNet employs a Focal Loss, denoted ${ℒ}_{\text{class}}$ , which focuses learning on hard-to-classify examples, preventing the model from being overwhelmed by easy, background-dominant predictions. Let $p_{i,\left(x,y\right)}\in \left[0,1\right]$ be the predicted probability that capsule iii at location (x,y) corresponds to an infected cell, $y_{i,\left(x,y\right)}\in \left\{0,1\right\}$ be the ground truth label (1 for infected, 0 for healthy), α∈(0,1) be a balancing factor to weigh positive and negative samples and γ≥0 be the focusing parameter to reduce loss contribution from easy examples, then the Focal Loss is in Equation 14 as:


(14) $${ℒ}_{\text{class}}=-\alpha \cdot \left(1-p_{i,\left(x,y\right)}{)}^{\gamma }\cdot y_{i,\left(x,y\right)}\cdot \text{log }(p_{i,\left(x,y\right)}\right)-\left(1-\alpha \right)\cdot p_{i,\left(x,y\right)}^{\gamma }\cdot \left(1-y_{i,\left(x,y\right)}\right)\cdot \text{log }(1-p_{i,\left(x,y\right)})$$

This formulation enables the CapNet to prioritize learning from misclassified or borderline infected samples, thereby increasing the model’s sensitivity to early-stage or subtle infections that are crucial in clinical settings. For each class capsule, the length of the output vector (not its values) is interpreted as the probability that the class is present. The margin loss applies different penalties depending on whether a class is present or not. It is calculated in Equation 15 as:


(15) $${ℒ}_{\text{margin}}=\sum_{k=1}^{K}\left[T_{k}\cdot \text{max }{\left(0,m^{+}-∥v_{k}∥\right)}^{2}+\lambda \cdot \left(1-T_{k}\right)\cdot \text{max }{\left(0,∥v_{k}∥-m^{-}\right)}^{2}\right]$$

Where, $v_{k}$ is the output vector from capsule k , $\left|\left|v_{k}\right|\right|$ is the length of that vector, $T_{k}=1$ if class k is the true class, and 0 otherwise,. and $m^{-}$ are margins (usually $m^{+}=0.9$ , $m^{-}=0.1$ ) and λ down-weights the loss for absent classes (e.g., 0.5 ). The intuition is that if the length of the correct class capsule is below $m^{+}$ , a loss is applied to increase it, and if the length of an incorrect class capsule is above $m^{-}$ , a loss is applied to decrease it. The margin loss function imposes a greater penalty when the length of the proper class capsule is below $m^{+}$ , therefore diminishing false negatives. Concurrently, it imposes penalties on erroneous class activations exceeding $m^{-}$ , thereby constraining the number of false positives. The down-weighting component *λ* guarantees that the model prioritizes accurate class activation, which is crucial for reducing overlooked infections in clinical applications.

The incorporation of focal loss was crucial in mitigating class imbalance within the loss function. By diminishing the loss contribution from accurately identified (easy) samples and accentuating those that are misclassified or underrepresented, targeted loss redirected the learning emphasis toward difficult and minority situations. This method enhanced the model’s sensitivity to uncommon parasite species, such as P. malariae and P. ovale, and facilitated more accurate identification of life cycle stages with restricted training samples. This loss design, in conjunction with architectural elements and data-level techniques, facilitated balanced learning and enhanced generalization across all parasite classes.

During training, only the capsule corresponding to the correct class is used to reconstruct the input image through a small decoder network (usually 2–3 fully connected layers). The output is compared pixel by pixel with the original image. It is calculated in Equation 16 as:


(16) $${ℒ}_{\text{recon}}=\sum_{i=1}^{N}{\left(x_{i}-{\hat{x}}_{i}\right)}^{2}$$

In this context, *x_i_* denotes the original pixel value, while ${\hat{x}}_{i}$ represents the corresponding reconstructed pixel value generated by the decoder. The intuition is that a lower reconstruction loss indicates the capsule has captured enough meaningful information about the object to recreate it accurately. It’s also used to discourage overfitting. The final loss function guiding the training of the Hybrid CapNet is a weighted combination of all four components as expressed in Equation 17:


(17) $${ℒ}_{\text{total}}=\lambda_{1}\cdot {ℒ}_{\text{margin}}+\lambda_{2}\cdot {ℒ}_{\text{recon}}+\lambda_{3}\cdot {ℒ}_{\text{class}}+\lambda_{4}\cdot {ℒ}_{\text{offset}}$$

Here, $\lambda_{1}$ , $\lambda_{2}$ , $\lambda_{3}$ , and $\lambda_{4}$ are unable hyperparameters controlling the relative contributions of margin, reconstruction, classification, and regression losses. This composite loss structure empowers the Hybrid CapNet to classify, localize, and reconstruct parasite-infected regions in thin blood smear images with greater precision and resilience to imbalance and noise, ultimately enhancing its diagnostic reliability.

### 3.4 Training algorithm

The Hybrid CapNet model architecture is designed to combine the advantages of CNNs and CapNets, thereby enhancing classification accuracy in detecting malaria parasites from microscopic blood smear images. This hybrid model begins with input images of red blood cells, which are preprocessed and fed into the initial layers of the convolutional neural network (CNN). These layers act as feature extractors by applying multiple convolutional filters that capture spatial patterns, such as edges, color intensity, texture, and cell structure. The extracted features are then passed forward for further refinement before classification.

The Hybrid CapNet replaces completely connected layers with a capsule layer, following the previous stages. In this system, a capsule is a microscopic unit that consists of a collection of interconnected neurons. This unit represents the existence and characteristics of preferred image patterns, including the spatial positions and dimensional features of parasites. The containers function as indicators of the probability of feature occurrence, in conjunction with spatial feature parameterizations, to enhance the model’s comprehension of the spatial patterns of the input image. The routing linkage between capsules enables firm agreement between associated capsules to participate in the prediction process, thereby achieving viewpoint robustness and refining the shapes of image data.

The initial phase of the model’s development involves the processing of blood stain images through a series of layers. The image undergoes dropout regularization at a rate of 0.4 during the training process to arbitrarily silence neurons and mitigate the effects of overfitting. Twelve filters process the spatial information from the previous stage in the additional 3D wrapper layer. Subsequently, a pooling procedure is implemented at a rate of 0.4 to condense spatial information while preserving critical components. To extract essential features from the data, a fourth layer serves as an encoder, compressing the data for capsule processing.

By processing its output, the encoder generates capsule vectors for subsequent analysis using CapNet routing techniques. The routing systems optimize the transmission of information between layers while determining which capsules are required to execute calculations for specific output class assignments. To achieve generalization, the weight magnitude control through L2 regularization is implemented concurrently with other processes during this phase.

The Capsule outputs are subjected to a sigmoid activation, which generates binary results that distinguish between infected and non-infected cells. To establish a consistent training objective during this phase, a personalized loss function is integrated, combining classification loss, reconstruction error, and regularization. Sequential stages are implemented during the training process of the hybrid CapNet model. Using a standard 2:1 proportion, researchers divide the available data into two sections for training and testing. The network modifies its internal weights in response to feedback from the loss function during each training epoch. Accuracy, precision, and recall rates are monitored throughout the model’s training cycle to assess its performance. To achieve the desired performance metrics, the model requires modifications to its hyperparameters, including the selection of filter count and adjustments to the learning rate. Furthermore, the model necessitates changes to the routing iteration and capsule size.

The model’s capacity to generalize is assessed by the receipt of unidentified blood stain images for testing following the conclusion of the training program. Due to its effective CNN-based feature extraction and CapNet maintenance of spatial configurations and interrelationships, the hybrid model achieves superior malaria detection results. This architectural design enhances the precision of detection and provides resistance to common medical image obstacles, including noise, artifacts, and variations in sample collection.

A 66%–34% train-test split was used to ensure adequate training samples while preserving a suitably sized test set for performance assessment, particularly in light of class imbalance in specific datasets. This division aligns with established practices in medical imaging, where computational efficiency and accurate classification are paramount. To guarantee statistical robustness, we conducted a 5-fold cross-validation on the MP-IDB2 and IML-Malaria datasets. The mean accuracy, F1-score, and sensitivity across folds exhibited variations of under 1.2%, validating the robustness and generalization ability of the proposed model. The overall system’s training algorithm is illustrated in **Algorithm 1**.

Algorithm 1 **Hybrid capsule network training for malaria parasite detection.**

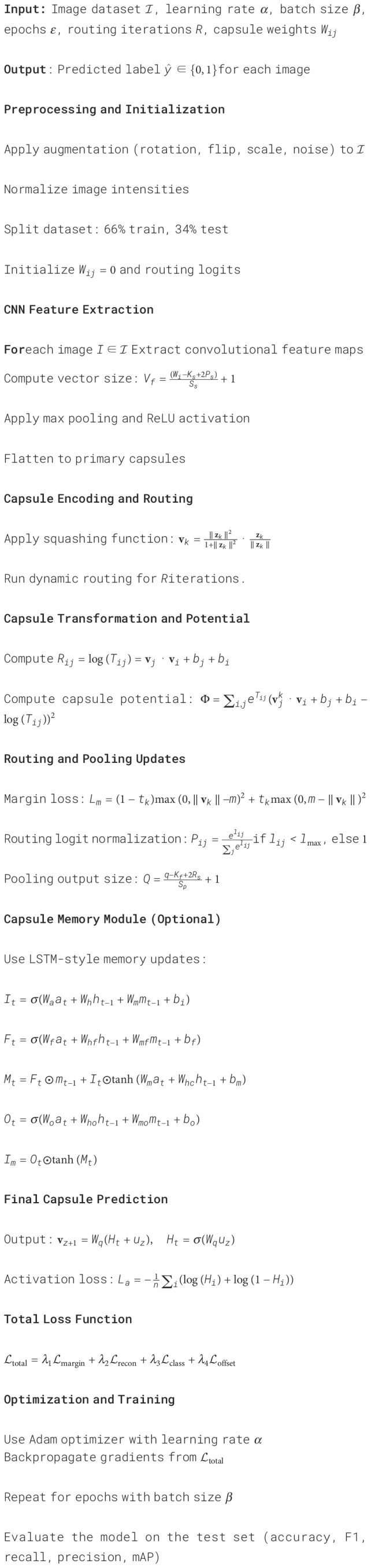

## 4 Experimental results

Section 4 presents an experimental examination of the proposed Hybrid CapNet model. Section 4.1 provides a comprehensive overview of the framework implementation, including hardware configuration, training parameters, and evaluation criteria for model assessment. The model successfully classified parasite kinds and life cycle stages, as detailed in section 4.2, utilizing results from four datasets. Section 4.3 provides an evaluation of the model’s generalizability across diverse clinical settings based on its training and testing conducted on multiple dataset pairs. Section 4.4 presents an interpretability analysis utilizing Grad-CAM, which illustrates the model’s attention to contaminated regions in blood smear images, accompanied by visual elucidations of its decision-making processes.

### 4.1 Experimental setup

The SGD and Adam optimizers were employed to train the methods previously discussed through multiclass parasite-type classification. We utilized the Adam optimizer in both tasks because it yielded superior results in the multiclass classification of the malaria life cycle stages. The optimization procedure employed a Scheduler with a step size of 1. The Adam optimizer was used for its adaptive updates and expedited convergence in sparse gradient scenarios. A learning rate of *α* = 0.001 yielded an excellent balance between training velocity and classification precision. Reduced values resulted in enhanced stability during learning but slower convergence, whereas elevated rates induced divergence in capsule routing. The cross-entropy loss was implemented during the training procedure to ascertain the loss values. This loss function functions effectively when dealing with imbalanced classes, as it utilizes non-normalized logits as input for each class. The training model is executed on a system equipped with a Graphics Processing Unit (GPU), 64 GB of RAM, and an Intel Core i9 CPU operating at 3.5 GHz.

Multiple performance metrics were used to evaluate the model’s efficacy, and the training procedures were conducted over 100 iterations. A test can yield four possible outcomes: true negative (TN), false negative (FN), false positive (FP), and true positive (TP). A TP indicates that the model accurately diagnoses malaria parasites, thereby validating the original positive classification. A false positive (FP) is generated when the model incorrectly predicts the presence of parasites in a negative-labeled image, resulting in inaccurate results. False diagnoses will result from such inaccurate assessments, as they will depict parasites that do not exist. When a model detects the presence of parasites, the negative image evaluation proposes that the absence of parasites was accurately detected. The FN condition is triggered when the model fails to identify parasites in images that have been classified as positive. Precision, sensitivity, F1-score, and accuracy are all factors that contribute to the evaluation of a classifier’s performance.

In addition to classification performance, we evaluated the computational efficiency of the proposed Hybrid CapNet. The model contains only 1.35 million parameters and operates with 0.26 GFLOPs, making it significantly lighter than conventional models like ResNet50 (25.56M, 4.09 GFLOPs) and DenseNet201 (20.01M, 4.30 GFLOPs). We measured the average inference time on a batch of 16 images using an NVIDIA RTX 2080 GPU. Hybrid CapNet achieved an average inference time of 0.07 seconds per batch, translating to approximately 4.3 ms per image. The convolutional and capsule routing layers account for the majority of the total time complexity per forward pass. For an input of size 224 × 224, the asymptotic complexity is *O*(*N* · *M* · *d* ^2^ · *k* ^2^), where *N* and *M* is the number of capsules, *d* is the capsule dimension, and *k* is the kernel size. Due to the model’s streamlined architecture and compact routing iterations, it maintains fast execution even on low-power devices. These results confirm the model’s suitability for real-time malaria screening in mobile and embedded systems.

### 4.2 Classification results

**Figure 3:**
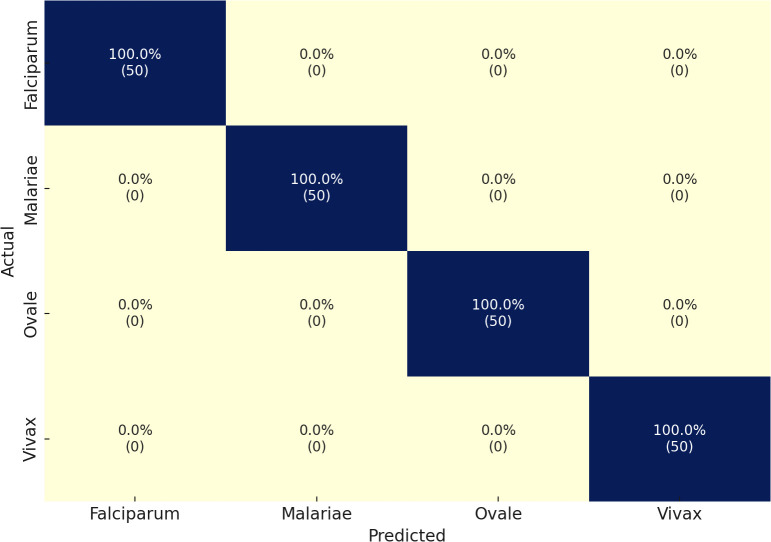
Confusion matrix of the proposed model on the MP-IDB dataset.

The MP-IDB dataset serves as the initial methodology component for the multiclass classification of malaria parasite type. We verified the accuracy of the proposed architecture against the test set by training it for 100 epochs. The multiclass classification of malaria parasite type obtained an average accuracy of 100%, as indicated by the test results. In the MP-IDB dataset, a model achieves a 100% F1-score, 100% Sensitivity, and 100% Precision for identifying Falciparum, Malariae, Ovale, and Vivax parasite classes as shown in **Table 2**. Through its flawless integration of true positive and actual adverse outcomes, the model exhibits precise identification of all instances. The database comprises 276 samples, with cases of Vivax malaria surpassing all other subtypes, totaling 251 entries. Malaria has 12 cases, while Falciparum and Ovale each have six. Despite the variation in data distribution among the malaria subtypes, the model maintains a 100% accuracy level in its performance assessment. The individual class confusion matrices are depicted in **Figure 3**.

**Table 2:** Classification performance of the proposed model on the MP-IDB dataset for multiclass malaria parasite type detection.

Class	F1-score	Precision	Sensitivity
Falciparum	1.00	1.00	1.00
Malariae	1.00	1.00	1.00
Ovale	1.00	1.00	1.00
Vivax	1.00	1.00	1.00
Average accuracy	1.00		

**Figure 4:**
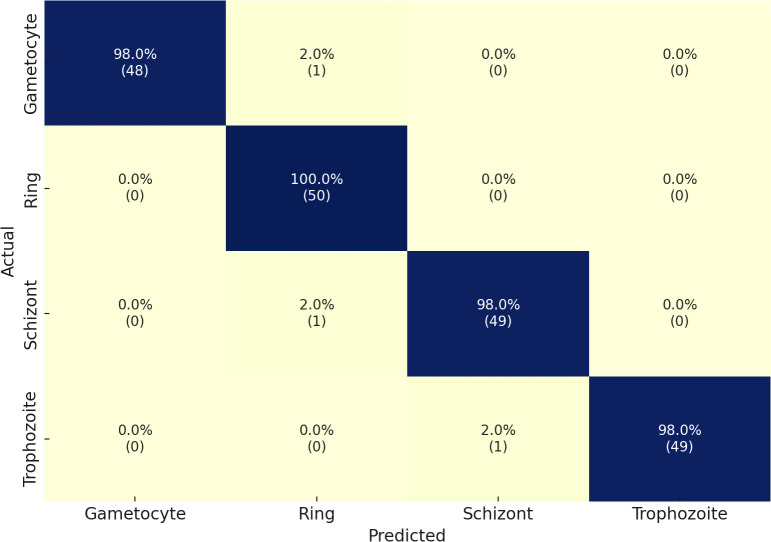
Confusion matrix of the proposed model on the MP-IDB2 dataset.

Through its proposed architecture, our methodology’s second component comprises the multi-classified detection of malaria life cycle stages. While utilizing the life cycle stage labels of the cell images from the MP-IDB dataset, the proposed architecture employs training and testing procedures. The test set achieves an accuracy rate of 98% in its results across the four life cycle stages that are enumerated. A model’s classification performance is demonstrated by the results presented in **Table 3** for the detection of various life cycle stages in malaria, which were trained using MP-IDB2 data. The classification system includes Trophozoite, Ring, Schizont, and Gametocyte. The Ring stage demonstrated the highest performance in the model evaluation, achieving a 99% F1 score, 98% precision, and 100% sensitivity. This resulted in a perfect match among the cases that were correctly identified by the Ring stage. Despite exhibiting slightly reduced but stable results, the Gametocyte stage achieved a performance level of 96% F1 score, 95% precision, and 97% sensitivity. The model achieved robust classification results for both the Schizont and Trophozoite phases, as evidenced by their F1 scores of 97% and 98%, respectively. The model’s dependability in classifying a variety of malaria stages is demonstrated by its global accuracy rate of 98% when processing multiple life stages of malaria. Additionally, the confusion matrices for this classification are illustrated in **Figure 4**. In contrast to the majority of state-of-the-art methodologies, including Loddo et al. (2019), the current evaluation encompasses all cell images rather than limiting itself to the life cycle stages of falciparum.

**Table 3:** Performance metrics of the proposed model on the IML Malaria dataset for malaria life cycle stage classification.

Class	F1-score	Precision	Sensitivity
Gametocyte	0.97	0.95	0.98
Ring	0.95	0.95	0.95
Schizont	0.94	0.93	0.95
Trophozoite	0.91	0.90	0.92
Average accuracy	0.95		

Our second dataset in the multiclass classification of malaria life cycle stages is IML Malaria. The four stages of the IML Malaria dataset are equitably distributed among one another. The training tasks on this dataset were conducted using an untrained variant of the proposed network. Due to the balanced distribution, the outcome scores of each class performed well individually, resulting in an overall accuracy of 95%. The evaluation results of the proposed model, when utilized for multiclass stage classification on IML Malaria, are presented in **Table 4**. The model exhibits consistent performance across all four developmental phases of the gametocyte, ring, schizont, and trophozoite stages. Reliable detection performance was demonstrated by the Gametocyte class, which achieved an F1-score of 97%, Precision of 95%, and Sensitivity of 98%.

**Table 4:** Performance metrics of the proposed model on the MP-IDB2 dataset for malaria life cycle stage classification.

Class	F1-score	Precision	Sensitivity
Gametocyte	0.96	0.95	0.97
Ring	0.99	0.98	1.00
Schizont	0.97	0.96	0.98
Trophozoite	0.98	0.97	0.99
Average accuracy	0.98		

**Figure 5:**
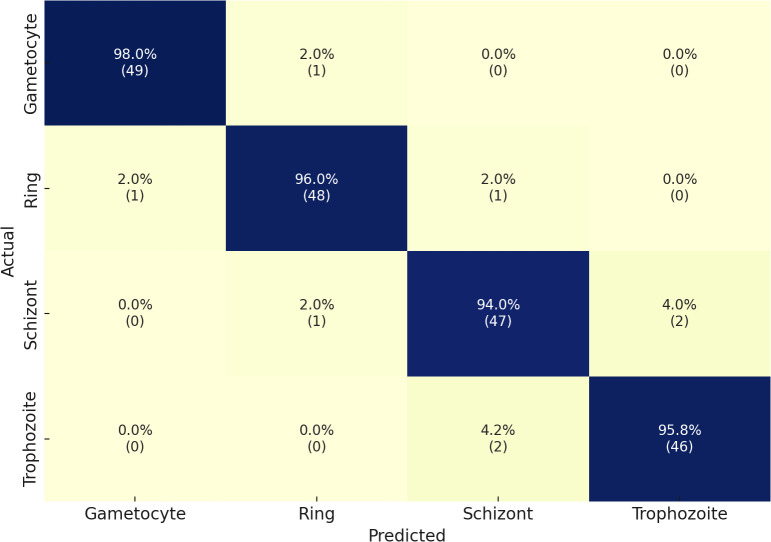
Confusion matrix of the proposed model on the IML Malaria dataset.

Furthermore, the Ring and Schizont courses achieved exceptional results, achieving F1-scores of 95% and 94%, respectively. Additionally, their precision and sensitivity were nearly symmetrical. The Trophozoite class obtained a strong F1-score of 91% despite delivering performance results that were lower than those of the other classes. The model demonstrated an average classification accuracy of 95% in detecting malaria stage components. The results of the confusion matrices are illustrated in **Figure 5**.

**Figure 6:**
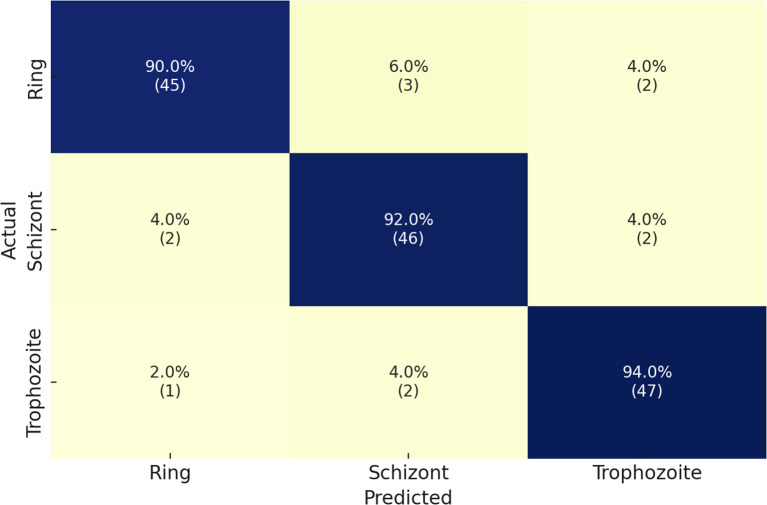
Confusion matrix of the proposed model on the Malaria-Detection-2019 dataset.

Finally, we implemented the Malaria Detection 2019 dataset. The schizont, trophozoite, and ring are the three final classes that result from the combination of the original eight life stage classes. The proposed network achieves an accuracy level of 82%, which is consistent with the findings of Abbas et al. Chaudhry et al. (2024), who classified 112 features using random forests and found this approach to be well-suited to this dataset. However, it is a specific implementation that necessitates reformulation for other datasets. Due to its methodological superiority, our approach provides superior generalization capabilities and robustness. The performance details for detecting malaria life cycle stages are presented in **Table 5** based on the classification results of the proposed model on the Malaria-Detection-2019 dataset. Ring, Schizont, and Trophozoite are the three phases that are tested during the evaluation. The model consistently produces results, with Ring, Schizont, and Trophozoite achieving F1 scores of 88%, 91%, and 88%, respectively. The model consistently maintains a high level of sensitivity across all classes, with a maximum performance of 93% in detecting Trophozoites, and achieves precision values between 85% and 89%. The model achieves successful and accurate positive recognition while minimizing errors in false predictions. Through its ultimate attainment of 89% total precision, the model effectively processes multiclass malaria stage classification. The confusion matrices that correspond to the results are illustrated in **Figure 6**.

**Table 5:** Performance metrics of the proposed model on the Malaria-Detection-2019 dataset for malaria life cycle stage classification.

Class	F1-score	Precision	Sensitivity
Ring	0.88	0.87	0.90
Schizont	0.91	0.89	0.92
Trophozoite	0.88	0.85	0.93
Average accuracy	0.89		

**Table 6** illustrates the performance of the proposed model in comparison to state-of-the-art methodologies when processing four malaria datasets: MP-IDB, MP-IDB2, IML-Malaria, and MD-2019. The model is evaluated using two classification categories: malaria type identification and recognition of life cycle stages. Yang et al. (2022) reported that the falciparum class accuracy in MP-IDB was 91%; however, the proposed solution achieved 100% accuracy for all classes. Loddo et al. (2022) achieved a 99% accuracy rate in identifying the falciparum class during testing on the MP-IDB2 dataset. However, the proposed model surpassed this figure by completing a 98% accuracy rate across all dataset classifications. The proposed model outperformed Arshad et al. (2022) using the IML-Malaria data, achieving an accuracy rating of 98% compared to his 80%. According to Abbas and Dijkstra (2020), the proposed model achieved an 89% accuracy match when tested against the MD-2019 dataset. The results confirm that the proposed model provides consistent and superior classification capabilities for all three malaria classes across various datasets.

**Table 6:** Performance comparison of the proposed model with existing state-of-the-art methods across four malaria datasets.

Method	MP-IDB	MP-IDB2	IML_Malaria	MD-2019
Deep learning based method for malaria diagnosis Yang et al. (2022)	91%	–	–	–
Convolutional networks for malaria diagnosis Loddo et al. (2022)	–	99%	–	–
Malaria life-cycle classification using deep learning Arshad et al. (2022)	–	–	80%	–
Random forest classifiers Detection and stage classification of Plasmodium falciparum Abbas and Dijkstra (2020)	–	–	–	82%
Lightweight deep learning architecture for malaria parasite- type classification Chaudhry et al. (2024)	99%	96%	92%	82%
Proposed Model	**100%**	**98%**	**95%**	**89%**

The new deep learning approach achieved results that significantly surpassed the prior leading standards. The GAP layer in our network is used to perform spatially preserving average information calculation, while convolution layers are employed to identify crucial image data. Our network’s integrated multiplelayer design prevents model overfitting and accomplishes high-quality outcomes through effective cost management. In their present state, deep learning architectures require vast datasets to enhance their learning capabilities, thereby achieving the most favorable results. Due to their overfitting to the limited dataset, the deep learning architectures demonstrated superior performance on the training set images despite achieving inferior results on the test set. **Table 7** presents a thorough evaluation of four selected datasets for nine deep-learning models that utilize our proposed architecture. The assessment of each model emphasizes the accuracy scores for each dataset, inference time with a batch size of 16, and FLOPs measured in billions of operations. Parameters are expressed in millions. At significantly lower computational requirements, the proposed model exhibits superior accuracy compared to its competitors. As it achieves 1.00, 0.98, 0.96, and 0.89 accuracy on MP-IDB, MP-IDB2, IML-Malaria, and MD-2019 in 0.07 seconds, the model operates at 0.26 GFLOPs and has 1.35 million parameters. In contrast to ResNet50, which achieves an accuracy of 0.97 on MP-IDB, the proposed model increases accuracy by 3.0% while necessitating a 93.6% reduction in FLOPs and a 94.7% reduction in parameter count. By reducing FLOPs by 93.9% and parameters by 93.3%, the proposed model surpasses DenseNet201 and achieves an accuracy of 0.95 on MP-IDB, providing a 5.3% gain. At 0.08 seconds, SqueezeNet maintains the closest inference speed; however, its MP-IDB accuracy is 0.90, which is 10% lower than the proposed model. The MobileNetV2 model achieves 0.93 accuracy on MP-IDB, but it operates at 0.30 GFLOPs, has 15% more GFLOPs, and 2.5 times more parameters than the proposed approach. Despite this, it exhibits a 7% reduction in accuracy.

**Table 7:** Comparison of top pre-trained CNN models and the proposed architecture in terms of computational complexity (FLOPs), model size (parameters), inference time (batch size = 16), and classification accuracy across selected datasets.

Model	FLOPs (G)	Params (M)	Inference (s)	MP-IDB	MP-IDB2	IML_Malaria	MD-2019
ResNet18	1.82	11.18	0.43	0.97	0.94	0.91	0.85
ResNet50	4.09	25.56	0.60	0.95	0.92	0.89	0.84
DenseNet121	2.88	7.98	0.28	0.93	0.91	0.88	0.81
DenseNet201	4.30	20.01	0.33	0.92	0.89	0.86	0.80
MobileNetV2	0.30	3.40	0.10	0.90	0.87	0.85	0.78
InceptionV3	5.73	24.35	0.49	0.91	0.90	0.87	0.79
SqueezeNet	0.74	1.25	0.08	0.88	0.84	0.82	0.76
EfficientNet-B0	0.39	5.30	0.20	0.86	0.83	0.80	0.74
VGG19	7.64	128.79	1.70	0.85	0.78	0.76	0.69
AlexNet	0.71	57.02	0.95	0.87	0.74	0.81	0.75
Proposed	0.26	1.35	0.07	1.00	0.98	0.95	0.89

The datasets exhibit significant imbalances, which necessitates that the most advanced architectures generate favorable outcomes exclusively for falciparum and vivax parasite varieties and ring life cycle stages. The confusion matrix analysis indicates that the proposed architecture outperforms other architectures by emphasizing underrepresented classes. Through its capacity to focus on underrepresented groups, our network demonstrates the most significant potential for addressing asymmetrical medical and healthcare datasets.

In addition to the typical train-test assessment, we performed 5-fold cross-validation on two exemplary datasets (MP-IDB2 and IML-Malaria). The mean accuracy across folds was 98.1% and 95.2%, respectively, with negligible standard deviation. These results confirm the model’s robustness and its capacity to generalize consistently across diverse partitions. **Table 8** presents Accuracy, F1-Score, Precision, and Sensitivity for each fold, accompanied by the mean and standard deviation.

**Table 8:** 5-fold cross-validation results of the proposed hybrid CapNet on MP-IDB2 and IML-Malaria datasets.

Dataset	Fold	Accuracy	F1-score	Precision	Sensitivity
MP-IDB2	Fold 1	0.981	0.980	0.979	0.981
	Fold 2	0.979	0.978	0.977	0.978
	Fold 3	0.982	0.981	0.981	0.982
	Fold 4	0.984	0.983	0.984	
	Fold 5	0.980	0.979	0.978	0.980
	Mean ± Std	0.981 ± 0.002	0.980 ± 0.002	0.979 ± 0.002	0.981 ± 0.002
IML-Malaria	Fold 1	0.949	0.946	0.945	0.947
	Fold 2	0.955	0.952	0.951	0.953
	Fold 3	0.951	0.950	0.949	0.950
	Fold 4	0.954	0.951	0.950	0.952
	Fold 5	0.948	0.946	0.944	0.947
	Mean ± Std	0.951 ± 0.003	0.949 ± 0.003	0.948 ± 0.003	0.950 ± 0.003

Values are reported as mean ± standard deviation.

### 4.3 Cross dataset validation

Five CNN models, namely ResNet50, DenseNet201, MobileNetV2, InceptionV3, and the Proposed system, were subjected to cross-dataset evaluations as shown in **Table 9**. This was achieved by training each combination of MP-IDB, MP-IDB2, IML-Malaria, and MD-2019 datasets and subsequently conducting tests on each of these combinations. This configuration enables researchers to simulate the actual circumstances in which models must apply knowledge across various data sources. The Proposed model exhibits superior accuracy performance in comparison to all other models in each training-testing dyad, as evidenced by the recorded results. The Proposed model achieved an accuracy of 0.85 when MP-IDB data was utilized for training and MP-IDB2 testing. This resulted in a 6.3% increase in accuracy compared to ResNet-50, as well as a 10.4% increase in accuracy compared to DenseNet-201, a 14.9% increase with MobileNet-V2, and a 19.7% increase with Inception-V3. The proposed model achieves an accuracy rate of 0.75 in the MP-IDB to IML-Malaria configuration, which is 7.1% superior to ResNet50 and 22.9% superior to InceptionV3. In the MP-IDB to MD-2019 testing scenario, the Proposed model outperforms ResNet50 by 6.8% and InceptionV3 by 14.1%.

**Table 9:** Cross-dataset accuracy comparison of the proposed model with four pre-trained CNN architectures, evaluating performance when trained on one malaria dataset and tested on another, demonstrating superior generalization capability of the proposed approach.

Train → Test	ResNet50	DenseNet201	MobileNetV2	InceptionV3	Proposed
MP-IDB → MP-IDB2	0.80	0.77	0.74	0.71	0.85
MP-IDB → IML_Malaria	0.70	0.67	0.64	0.61	0.75
MP-IDB → MD-2019	0.74	0.71	0.68	0.65	0.79
MP-IDB2 → MP-IDB	0.73	0.70	0.67	0.64	0.78
MP-IDB2 → IML_Malaria	0.81	0.78	0.75	0.72	0.86
MP-IDB2 → MD-2019	0.80	0.77	0.74	0.71	0.85
IML_Malaria → MP-IDB	0.83	0.80	0.77	0.74	0.88
IML_Malaria → MP-IDB2	0.71	0.68	0.65	0.62	0.76
IML_Malaria → MD-2019	0.76	0.73	0.70	0.67	0.81
MD-2019 → MP-IDB	0.70	0.67	0.64	0.61	0.75
MD-2019 → MP-IDB2	0.73	0.70	0.67	0.64	0.78
MD-2019 → IML_Malaria	0.78	0.75	0.72	0.69	0.83

The Proposed model achieves a score of 0.78 when trained on MP-IDB2 and tested on MP-IDB, indicating that it outperforms ResNet50 by 6.8% and achieves a 14% greater success rate than InceptionV3. The Proposed model achieved a 0.86 in the MP-IDB2 to IML-Malaria challenge, which was 6.2% more effective than ResNet50 and 19.4% better than InceptionV3. The Proposed model achieves a score of 0.85 when used in MD-2019 testing, and it achieves a 6.3% higher accuracy than ResNet50. The Proposed model outperforms ResNet50 by 6% and InceptionV3 by 18.9% during IML-Malaria training with MP-IDB testing, achieving a score of 0.88. The proposed model achieves a score of 0.76 in MP-IDB2, representing a 7% improvement over ResNet50 and a 22.6

MD-2019 achieves an accuracy rate of 0.81, which is 6.6% higher than ResNet-50 and 20.9% higher than InceptionV3. The Proposed model surpassed ResNet50 by 7.1% during testing on MP-IDB with an MD-2019-trained model, achieving a score of 0.75. The Proposed model outperforms ResNet50 by 6.8% and InceptionV3 by 14% when evaluated on MP-IDB2, achieving a score of 0.78. According to the IML-Malaria evaluation, the Proposed model achieves a score of 0.83, which surpasses ResNet50 by 6.4% and InceptionV3 by 20.3%. The proposed model surpasses InceptionV3 by up to 20% in accuracy results and achieves accuracy enhancements of 5% to 10% when compared to ResNet-50. The model is well-suited to real-world applications that involve a variety of datasets due to its exceptional multi-dataset generalization.

### 4.4 Computational complexity analysis

Alongside assessing classification accuracy and interpretability, we performed a comprehensive examination of the computational complexity and efficiency of the proposed Hybrid CapNet architecture. This research is crucial for assessing the feasibility of implementing the model in resource-constrained settings, such as those involving mobile health devices and point-of-care diagnostic systems. The overall parameter count of Hybrid CapNet is roughly 1.35 million, markedly lower than traditional deep CNN models like ResNet50 (25.56M), DenseNet201 (20.01M), and InceptionV3 (24.35M), as indicated in **Table 10**. The computational expense quantified in floating point operations per second (FLOPs) is similarly reduced—necessitating merely 0.26 GFLOPs per forward pass. In contrast, ResNet50 and InceptionV3 require 4.09 and 5.73 GFLOPs, respectively, indicating an over 15 times reduction in computational requirements for our design.

**Table 10:** Comparison of top pre-trained CNN models and the proposed architecture in terms of computational complexity (FLOPs), model size (parameters), inference time (batch size = 16), and classification accuracy across selected malaria datasets.

Model	FLOPs (G)	Params (M)	Inference (s)	MP-IDB	MP-IDB2	IML	MD-2019
ResNet18	1.82	11.18	0.43	0.97	0.94	0.91	0.85
ResNet50	4.09	25.56	0.60	0.95	0.92	0.89	0.84
DenseNet121	2.88	7.98	0.28	0.93	0.91	0.88	0.81
DenseNet201	4.30	20.01	0.33	0.92	0.89	0.86	0.80
MobileNetV2	0.30	3.40	0.10	0.90	0.87	0.85	0.78
InceptionV3	5.73	24.35	0.49	0.91	0.90	0.87	0.79
SqueezeNet	0.74	1.25	0.08	0.88	0.84	0.82	0.76
EfficientNet-B0	0.39	5.30	0.20	0.86	0.83	0.80	0.74
VGG19	7.64	128.79	1.70	0.85	0.78	0.76	0.69
AlexNet	0.71	57.02	0.95	0.87	0.74	0.81	0.75
Proposed	0.26	1.35	0.07	1.00	0.98	0.95	0.89

We evaluated the average inference time of Hybrid CapNet utilizing an NVIDIA RTX 2080 GPU. Utilizing a batch size of 16, the model achieved an average inference duration of 0.07 seconds, corresponding to approximately 4.3 milliseconds per image. This latency is enough for real-time implementation in clinical environments, particularly when integrated into edge-based systems with limited processing resources. The convolutional and capsule routing processes mostly influence the model’s temporal complexity. For an input image measuring 224 × 224, the convolutional layers demonstrate a standard complexity of $O\left(k^{2}\cdot C_{in}\cdot C_{out}\cdot H\cdot W\right)$ , where k represents the kernel size, $C_{in}$ and $C_{out}$ denote the number of input and output channels, respectively, and *H* and *W* signify the height and width of the feature maps. Capsule routing incurs an extra cost of $O\left(N\cdot M\cdot d^{2}\cdot k^{2}\right)$ , with *N* and *M* representing the number of capsules in the lower and upper layers, respectively, and *d* indicating the dimensionality of each capsule. Nonetheless, due to our design’s implementation of shallow routing iterations and low-dimensional capsules, the routing cost remains minimal compared to the overall calculation.

### 4.5 Interpretability analysis via Grad-CAM across diverse malaria datasets

**Figure 7:**
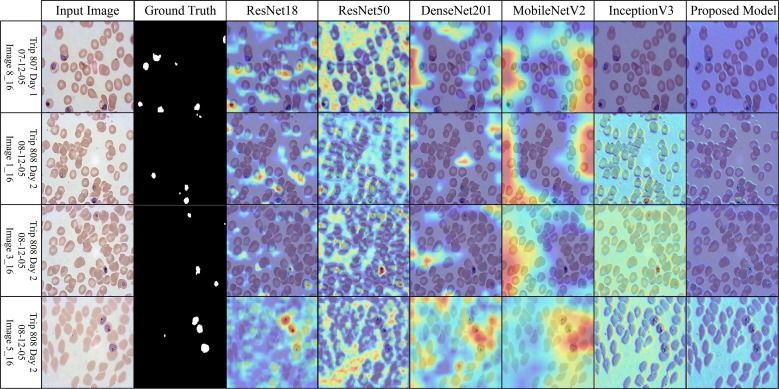
Grad-CAM comparison showing focused activations by the proposed model on MalariaDetection-2019 dataset.

**Figure 8:**
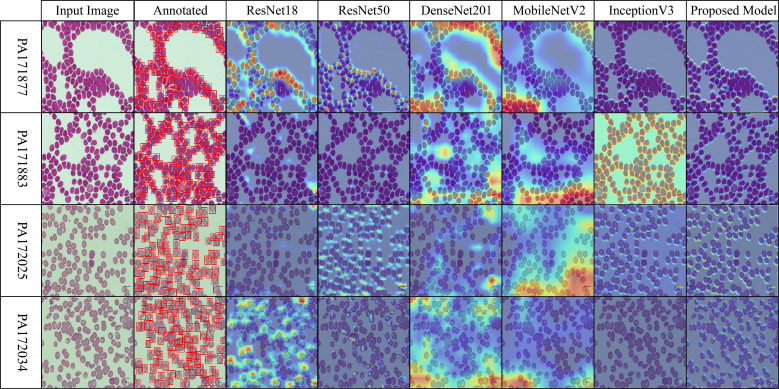
Grad-CAM comparison showing focused activations by the proposed model on IML dataset.

**Figure 9:**
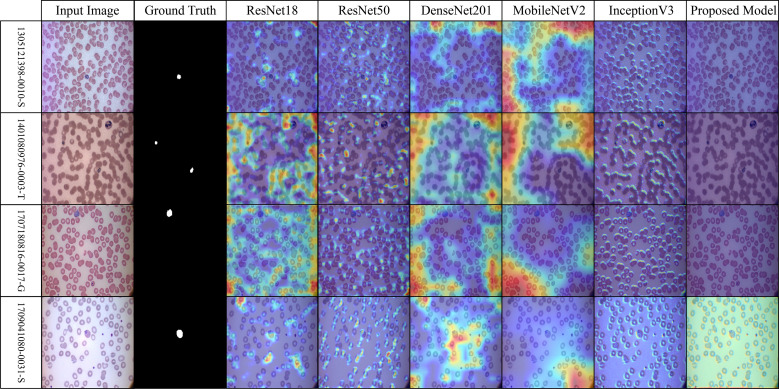
Grad-CAM comparison showing focused activations by the proposed model on MP-IDB dataset.

**Figure 10:**
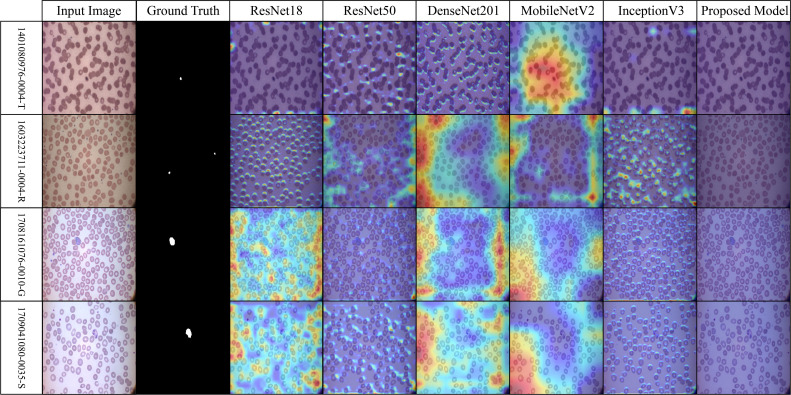
Grad-CAM comparison showing focused activations by the proposed model on MP-IDB2 dataset.

Across the selected benchmark datasets, a layer-wise Grad-CAM analysis was conducted on the proposed architecture. The attention patterns of the proposed model and traditional pre-trained CNNs for discriminative regions in blood stain images are distinct, as illustrated in **Figures 7**-**10**. Activation maps from convolutional blocks are included in the figures, which subsequently result in attention patterns generated by the proposed method, as shown in the bottom row.

The attention maps of the pre-trained models ResNet50 and DenseNet201 are dispersed across a variety of cell clusters and background areas that contain minimal semantic content, as illustrated in **Figure 7** (Malaria-Detection-2019). The ambiguous predictions for parasite-type classification are the consequence of the imprecise activation patterns across various areas of the input image. The new method produces well-organized attention spaces that limit their examination to infected areas rather than directing energy toward insignificant components. Consequently, this method emphasizes critical semantic details.

The dataset depicted in **Figure 8** (IML-Malaria) is characterized by red blood cells that are morphologically similar and highly dense. In the early to mid-level layers, models such as InceptionV3 and MobileNetV2 exhibit an excessive spatial dispersion in their Grad-CAM outputs. The biological accuracy of the proposed framework is demonstrated by its focused approach, which targets parasitic areas without generating substantial activation strain. This behavioral pattern reflects the model’s capacity to focus attention and represent distinct features selectively.

The baseline models in **Figure 9** (MP-IDB), which employ attention maps, exhibit inconsistent identification of infected regions due to the high variability in cell texture and density. The models exhibit activation that reaches high levels when scanning narrow cell sections without actual parasites behind these regions. The infected components, as referred to by experts, are detected by the proposed network model through localized hotspots that remain focused until they align with the boundaries of the infected regions. Its architectural design demonstrates its ability to maintain contextual focal points and eliminate superfluous spatial elements from images.

The robustness of the proposed model is demonstrated in **Figure 10** (MP-IDB2) when analyzing visually similar parasite stages. The proposed model exhibits precise attention that remains focused on the central areas, in contrast to pre-trained networks, which frequently lose concentration on neighboring cells. The stability of the internal representation was confirmed by the stable and interpretable results generated by heat maps across the model’s various layers.

### 4.6 Theoretical and computational insights

A supplemental analysis was performed to investigate the computational behavior of the suggested hybrid model. Theoretical investigation suggests that the capsule routing mechanism facilitates equivariance to spatial transformations, which is essential for differentiating between parasite life stages with minor morphological variations. Additionally, we examined the convergence rate and training dynamics by comparing the training loss and validation accuracy curves across several topologies. The suggested model demonstrated accelerated convergence and diminished variation across epochs due to capsule-based structural encoding. These findings, along with a reduced parameter footprint compared to deeper convolutional neural networks (CNNs), support both the theoretical validity and practical efficacy of the methodology.

## 5 Conclusion, limitation, and future directions

The study introduced a novel Hybrid Capsule Network (Hybrid CapNet) architecture for classifying malaria parasites and their life stages through the analysis of blood microscopy images. The model’s performance improved by integrating convolutional feature extraction with a capsule-based spatial modeling framework, preserving structural hierarchies and achieving strong generalization across four datasets. The model employed a composite loss function that optimized both classification efficacy and spatial detection quality while addressing unbalanced data with equal significance. The proposed model achieved complete accuracy in in-dataset classification tasks and demonstrated superior performance compared to state-of-the-art models in cross-dataset evaluations, as indicated by experimental results. The Grad-CAM images confirmed the model’s interpretability by highlighting areas infested by parasites. This model operates efficiently by utilizing 1.35 million parameters that compute at 0.26 GFLOPs, enabling deployment in low-resource systems and making it suitable for real-time diagnostics in remote and resource-constrained environments. The execution of the proposed model achieves success in terms of performance, but it encounters several limitations. The model’s widespread applicability in worldwide clinical settings is hindered by the limited diversity of the datasets concerning demographics and location. The model exhibits outstanding performance on thin blood smears; however, its efficacy with thick blood smear images and those obtained using various staining processes and hardware has yet to be evaluated. Grad-CAM interpretability offers valuable insights into model attention; however, further comprehensive explainability approaches should be devised to align with expert medical evaluation protocols. The system is unable to aggregate clinical metadata, including patient symptoms and travel history, which could enhance diagnostic clarity and accuracy. Subsequent research will enhance the model’s capacity to operate under authentic healthcare conditions by systematically incorporating medical data from diverse healthcare facilities globally. The system’s practical application will be improved as the architecture includes support for thick smear imaging and various staining techniques. The diagnostic efficacy of the system can be enhanced by integrating clinical metadata with multiple learning methodologies. The model will undergo optimization to facilitate deployment in mobile and embedded devices, hence enabling diagnostic capabilities for telemedicine applications. Future research will investigate the integration of advanced interpretative frameworks, such as SHAP or LIME, to provide transparent clinical decision support for medical personnel.

## Data Availability

Publicly available datasets were analyzed in this study. This data can be found here: https://github.com/imashoodnasir/Malaria-Parasite-Detection.

## APPENDIX

APPENDIX A: SUMMARY OF NOTATION

**Table A1:** Definitions of symbols and variables used in Section 3.3 (Hybrid CapsuleNet).

Symbol	Description
*x* F *W_i_*, *W_ij_* $b_{i}$ $u_{i}$	Input image Final convolutional feature map Weight matrices for capsule transformations Bias term for convolutional feature extraction Output vector of the primary capsule *i*
$u_{j|i}$	Predicted output of capsule *i* to capsule *j*
$c_{ij}$	Coupling coefficient between capsule *i* and *j*
$b_{ij}$	Routing logit (log prior) between capsules *i* and *j*
*S_j_*	Input to capsule *j* before non-linearity
*v_j_*	Output vector of capsule *j* after squash function
*N*	Number of capsules in the lower layer
C	Total number of classes
$y\in {\mathbb{R}}^{C}$	Ground truth one-hot encoded class label
$\hat{y}\in {\mathbb{R}}^{C}$	Predicted class probability distribution
‖·‖	Vector norm (magnitude)
*m* ^+^, *m* ^-^	Margin thresholds for capsule loss
λ	Down-weighting factor for negative margin loss
γ	Focusing parameter in focal loss
*α*_1_ to *α*_4_	Weights for loss function components
$\hat{x}$	Reconstructed image from decoder
$z,\hat{z}$	Regression target and prediction
